# Proceedings of Second International conference on Medical Writing held at Ajman, UAE (March 5-7, 2015)

**DOI:** 10.12669/pjms.313.7978

**Published:** 2015

**Authors:** Shaukat Ali Jawaid

Ajman (UAE): EDC, Center for Diabetes Education in collaboration with World Association of Medical Editors (WAME), European Medical Writers Association (EPWA) and Eastern Mediterranean Association of Medical Editors (EMAME) organized a two day international conference on Medical Writing here from March 5-7, 2015.

Mr. Tom Lang from USA a distinguished Medical Writer was the first speaker in the first session on March 5, 2015. Tracing the history, he gave details of the formation of various professional association of medical, science editors. The International Committee of Medical Journal Editors (ICMJE) he said, came into being in 1968 in response to a letter from a Secretary to the Editors of NEJM, AIM and JAMA about creating a standard reference format for medical journals. A meeting was then held at Vancouver in 1978. At that time ICMJE was a small self appointed working group of editors from general medical journals. It used to meet annually and funded its own programme. It decided to make recommendations for the conduct, reporting, editing and publications of scholarly work in medical journals. Members of this group included Annals of Internal Medicine, BMJ, CMJA, Chinese Medical Journal, and Ethopian Journal of Health Sciences, JAMA, NEJM, Lancet, PLos and a few others. It was in 1979 that it released the Uniform Requirements for Manuscripts Submitted to Biomedical Journals. Since then it has issued important position papers that have changed how science is reported.

World Association of Medical Editors (WAME) was founded in 1995 with 22 medical journal editors and scientists from thirteen countries from five continents. It is an alternative to ICMJE which is small and exclusive. Today it has nineteen hundred members from over a thousand journals from ninety two countries. WAME membership is open to decision making editors of medical journals worldwide and to scholars in the field of scientific publishing. Its membership is free. WAME facilitates worldwide cooperation, exchange of educational information, improve editorial standards, promotes the professionalism, expand the influence of medical editors, develop mutual support, encourage research to improve the quality of medical science and practice. WAME support this concept of regional groups based on language and geography. It is a virtual organization. It has different committees on Ethics, Editorial Policy and Education.

Council of Science Editors (CSE) was founded in 1957 by the National Science Foundation and the American Institute of Biological Sciences as the Council of Biology Editors. Its name was changed to CSE in 2000 and today it has over eight hundred members. It publishes position papers on its website, offers journal editors certification programme and holds annual conferences with course for journal editors and manuscript editors.

European Association of Science Editors (EASE) was founded in 1982 in France by a merger of European Life Science Editors Association and the European Association of Earth Science Editors. At present it has over 550 members representing more than fifty countries. It publishes a quarterly journal European Science Editing; it has published Guidelines for authors and Translators of Scientific Articles and holds its conference annually.

Other organizations of Authors, Editors and Medical Writers include American Medical Writers Association (AMWA), European Medical Writers Association (EMWA), and International Society for Medical Publication Professionals (ISMAPP) and the industry sponsored The International Publication Planning Association (TIPPA).

Speaking about the formation of Eastern Mediterranean Association of Medical Editors (EMAME) Prof. Farhad Handjani President of EMAME said that it uses potential of people in the region. It also provides an opportunity for networking with people outside the region. We can have joint workshops on Medical Writing, Peer Review. At present EMAME represents over four hundred biomedical journals from the Region. Good research, good papers and good journals are all interlinked. There are certain ethical issues like publication ethics, cut and paste etc., because of pressure to publish on academicians. When there is too much pressure, people start working unethically, he remarked.

European Medical Writers Association Mr. Tom Lang said at present has 941 members from Europe, UK and Germany. It is a not for profit organization working with professionals, medical writers, editors, publishers. Its Flagship is workshops and so far it has organized over hundred workshops. Writing, Editing conferences are expensive. Webinars, e learning are being used effectively. The Medical Writing journal has good contents. Speaking about publication ethics, Tom Lang emphasized the importance of transparency. We the Medical Writers are often accused of ghost writing but we are ethical and professional people. There should be transparency when the services of professional medical writers are acquired. Medical Writers prepare ghost papers writing for Pharma industry. They are serving the pharmaceutical industry not science. Continuing Mr. Tom Lang said that professional medical writers are necessary, desirable as it saves the time and money. The authors have complete control on data. He called for close collaboration between the medial editor’s and medial writers.

Participating in the discussion Prof.Farhad Handjani said that professional medical writers are working in various countries but they are not active in our region. Professional medical writers attend workshops and put professionalism in their job. They also organize training courses. Prof. Farhad Handjani was of the view that our own people should also start writing as well. To encourage that we at the Shiraz University of Medical Sciences have started a Masters course in Medical Journalism. We need to open up this concept of professional medical writers in this region and the services of professional medial writes should be included in the acknowledgement section in the manuscript.

## Statistical Concepts in Clinical Research

**Mr. Tom Lang’s** next presentation was on statistical concepts in clinical research. He pointed out that most authors use only basic statistics if they use it at all. As regards underuse of statistical analyses, upto 80% of articles use only descriptive statistics, a vast majority use only very basic statistics while only 20% were using more advanced statistics methods. Yet another problem was that authors who use statistics make lot of mistakes hence there is a high rate of statistical errors in manuscripts. Almost 70% of articles reporting studied have statistical flaws. Statistical problems in the literature, he said, are widespread, potentially serious, largely unknown with the result that much of the scientific literature, he opined, cannot be trusted.

Continuing Mr. Tom Lang said that many people were not aware of the first two problems. When the standard deviation is greater than half the mean, the data is not normally distributed. He then referred to the CONSORT statement. Intermediate results, Mr. Tom Lang said falls between a negative result and positive result. It indicates neither a positive nor a negative finding. He emphasized the importance of using figures and tables accurately as readers remember the usual impression of the figure better than the actual data. All statistical tests have assumptions and if these assumptions are violated, one has less confidence in the results. A visual summary of the study can summarize the study design, show the number of patients at each stage, indicate denominators for proportions, percentage and present the main results of the study.

**Statistical problems in literature are widespread, potentially serious & largely unknown-Tom Lang**

## Programme for Diabetes Screening

**Prof. Jaakko Tuomilehto** from Finland talked about writing a programme for diabetes screening. Screening, it was stated is carried out for early detection of disease in individuals who do not show any signs of disease. Screening can be of various types i.e. Mass screening for whole population, selective screening of those at high risk besides multiphase screening wherein several diseases are screened at once while screening for surveillance is for repeated observations. The purpose of screening is to reduce morbidity and mortality. It is done with applying relatively simple inexpensive tests to people who are asymptomatic for the purpose of classifying them with respect to their likelihood of having a particular disease. Screening tests can also be used as diagnostic tests. Diagnosis involves confirmation of presence or absence of disease in someone suspected of or at risk for disease. Screening can be for prevalence of disease and susceptible to benefit from treatment or for risk of future disease and susceptible to benefit from preventive intervention. He then talked about the screening process in detail. Key measures of validity of screening tests are sensitivity, specificity, positive predictive value and negative predictive value. The proportion of all those who do not have the disease and who test positive are known as false positive and the proportion of all those who have the disease but test negative are known as false negative. Sensitivity is the ability of a test to identify those who have a disease while specificity is the ability of a test to identify those who do not have the disease.

Speaking about the considerations in screening Prof.Jaakko mentioned severity of the disease, its prevalence, understanding the natural history, diagnosis and treatment, cost, efficacy and its safety. The criteria for successful screening programme is the presence of the disease in the population being screened, high morbidity and mortality and it must be an important public health problem. In addition, early detection and intervention must improve the outcome. Natural history of the disease should be understood in such a way that the detectable sub-clinical disease stage is known and identifiable. It must also be ensured that the screening test is relatively sensitive and specific, should be simple and inexpensive, safe besides being acceptable to the subjects as well as the providers. It must also be able to offer something to those who screen positive. Facilities for diagnosis and appropriate treatment must be available. It is unethical to offer screening when no services are available for subsequent treatment.

**It is unethical to offer screening when no services are available for subsequent treatment-Prof.Jaakko**

Discussing the screening strategies Prof. Jaakko said that the high risk approach should be cost effective, intervention should be appropriate for the individuals, subjects should be motivated. However, it fails to deal with the root causes of the disease and there is small chance of reducing disease incidence at the population level. On the other hand population approach has the potential to alter the root causes of the disease, it also offers greater chance of reducing the disease incidence, offers small benefit to the individuals, there is poor subject motivation and there is the problematic risk-benefit ratio. Higher BMI means higher the incidence of diabetes, hence screening test is justified in this condition. There are certain risks of screening i.e. True positive are labeled as diseased and false positive can have side effects of diagnostic tests and treatment besides anxiety and monetary expenses. On the other hand false negative can delay intervention. There is disregard of early signs and symptoms which may lead to delayed diagnosis. Most diseases do not progress at same rate in every one. Screening will pick up slowly developing disease. Volunteers have better health, lower morbidity and they are likely to adhere to mediations prescribed. In order to find out whether a screening programme is effective, it should be evaluated afterwards but it is rarely done. Prof. Jaakko concluded his presentation by stating that assessing disease screening programme is complex. Proof of value of screening for type 2 diabetes is lacking but there is reasonable supportive evidence. Screening programmes for type 2 diabetes should start with simple self complete screening tools.

## Publication Ethics

Speaking in the workshop on publication ethics **Prof.Tom Lang** first referred to the ICMJE criteria for authorship and to be eligible for authorship, every individual must meet all the ICMJE listed criteria which are as under:

1. Contribution to the research
2. Contribution to preparing the manuscript
3. Approving the final version to be published
4. Agreeing to assist in any investigation of the research.

In addition he or she must be able to say which co-author is responsible for each part of the research and have confidence in the integrity of the contribution of all the co-authors.

For authors it is extremely important to meet all the four conditions. Just getting grants, referring patients, collecting data or supervising the research does not justify authorship. One should never allow guest or gift authorship to seniors, directors, those who provide patients, samples etc. Authors should be listed in order of greatest contribution to the work from most to the least contribution. It is advisable to decide about listing of authors even before starting the research. It is also essential to acknowledge interest, competing interest, personal financial academic interest. He also referred to outright fraud and citation amnesia. Methods for selection of sample may be there. It is a copy right issue, plagiarism but not fraud. Duplicate submission, simultaneous submissions were also discussed. Duplication publication Mr. Tom Lang opined has effect on practice of medicine as it affects the number needed to treat. Duplicate publication in a different language is allowed provided it is properly acknowledged. Salami slicing means cutting something into thinner pieces. Salami slicing is not a good thing. Article reporting different parts of the same research should indicate that they are all from the same study. Patients have a right to privacy. Image manipulation i.e. changing brightness and contrast on only part of the image, using cloning tools to hide details or cropping images to eliminate information is also included in publication misconduct. Journal of cell biology has started screening the images of all accepted papers since 2002.

Journal editors and peer reviewers are not supposed to tell anyone that they have reviewed a particular article or use the information in the article for any other purpose besides delaying their decision on a manuscript until a competing work is published. Authors, co-authors and reviewers are supposed to disclose their possible conflict of interest to the journal editor while the possible conflicts of interest among the authors are mentioned in the published manuscript.

Predatory journals, Mr. Tom Lang opined are those which offer quick acceptance, have no peer review and they usually inform authors of publication fee after acceptance. They use websites mimicking good reputed journals. He then also referred to Jeffrey Beall who publishes Beal’s list of black list of questionable open access journals. One should look at Directory of Open Access Journals which publishes a white list of legitimate online journals.

Plagiarism Mr. Tom Lang said means taking credit for the written or creative work of someone else. Science builds on the work of others so it is the intentional misattribution of the work that is plagiarism not necessarily the use of the work itself. Citation plagiarism or citation amnesia means not giving credit to other authors and letting readers assume that you or someone else deserves the credit. On the other hand outright fraud stands for theft of intellectual property. Self plagiarism means resubmitting large portions of one’s own writing without disclosing its earlier publication to the new editor. Usually it is a copy right issue. During the discussion the contributor ship now being practiced by various journals was also highlighted.

## Research Ethics in Publications

**Prof. Farhad Handjani** discussed research ethics in publications. He pointed out that while practice helps an individual, research benefits many. He mentioned about respect for persons, beneficence and justice. He opined that while doing research one has to think of the society. It was in 1964 that Helsinki declaration was made. It is important that negative results of research are also published. Conflict of interest should be declared. Side effect of some drugs are never published. He then talked about the role of Institutional Review Boards, Ethics Committees and said that ECs should consist of people with different background including lay people and scientists as well. IRBs must look that the risk is minimized, look at favorable risk benefit ratio of studies, equitable subject selection, and informed consent of the research subjects, data should be monitored for safety. Privacy of research subjects should be protected and there should be adequate safeguards for vulnerable individuals. Ethics Committee approval means the Editor cannot question legitimacy of the study. Informed consent of research individuals is very important. It is a process. Informed consent can be oral or written. One should talk to the village chief or community elders before undertaking research in their respective areas. One should also respect the cultural values. Volunteers should have the choice to come out of the study trial. In case the children and adolescents are involved in research, the assent should be given by their parents. Clinical Trials Registration, Prof. Farhad Handjani remarked is a must.

**IRBs must look at favorable risk benefit ratio of studies, equitable subject selection, & informed consent of the research subjects-Farhad Handjani**

On Day two of the conference **Julia Donnelly** from UK was the first speaker who discussed how to write a Good Poster and create an impact. Posters, she said, are enlarged, graphical presentation of research data. The abstract represents citable references and it also ensures rapid publication. The advantages of posters are that they can be viewed at leisure time, offers personal contact with the author. They are more comprehensive than an oral presentation. They can also be more memorable than a talk. Their value can be prolonged through handouts and it can also be a fun. However, it also has some disadvantages as the viewer is not comfortably seated. It is easy for the viewer to walk away. If a poster is dull, the viewer may switch off. It is time consuming to produce and at times it is also difficult to decide what to left out. Oral presentation is rated highly as compared to poster presentation. The writer should try to be brief.

If the poster is brief, structured, good appearance, has better title, lay out is good and conclusions are highlighted, it will have an impact. Most common complaints regarding posters is the use of too small type face or difficult to read, including too much un-necessary data, confusing organization, lack of headings and the information provided is not newsworthy. Some of the key steps in planning poster once the abstract has been accepted are to write the poster text. Have proper layout. Find out what does the abstract say, what results are available, who is your target audience, when the poster is to be presented. Speaking about general principles of poster text, she said that ensure maximum use of white space, remove non-essential information, use abbreviations, and use the active voice, use tables and figures to replace words, have short sentences and use bullets. Write in defined sections and there should be emphasis on results. Write Methodology and Results first. About 10% should be introduction, 15% methods, figures 2%, Results 30%, tables 2%, conclusions 10% and references 5%. Title should be clear, concise with less than ten words. For authorship follow ICMJE guidelines.

Introduction in poster should be brief and relevant, limit the information, ask pertinent questions, and use present tense. Poster has to be short and convey the message. In the methods section, include only essential information, describe design. State the results factually without commentary and interpretation, direct the reader to tables and figures and use past tense with short sentences. Poster contents should be brief and relevant. In references list first author and then et al. limit to relevant references, simplify tables, answer question posted in the introduction, give presenter contact with e mail and write poster number. Size of the poster should be as per instructions. Poster must be eye catching, be creative, use colours and unusual format, and locate tables and figures near relevant test, use upper and lower case type. For title use 30-36 point type font, for authors 30 point bold, heading 30 point, body text 20 point and not bold. While reviewing the poster detect errors, inappropriate and poor writing, improve its quality and increase the likelihood of good response. Make sure that the written piece is suitable for a poster, is it suitable for target audience, does it include the right message and does it have appropriate contents. Be clear, concise and avoid un-necessary details.

**Prof. Paolo Pozzilli** from UK discussed pros and cons of peer reviewing. Peer review of a paper, it was stated, can be arranged in your own institution. It is much more critical and dynamic process than many other forms of quality regulation. Peer review is important because it helps determine whether a study’s conclusions follow logically from the procedures used to arrive at it and whether the conclusions makes a significant contribution to our knowledge. Unintentional plagiarism means paraphrasing poorly, changing a few words without changing the sentence structure of the original or changing the sentence structure but not the word, quoting poorly. On the other hand intentional plagiarism means passing off as one’s own pre-written papers from other sources., cut and paste and borrowing others ideas. Ethical medical writers must always acknowledge the original source of the idea, text or illustration. Integrity of clinical trials, it was emphasized, is essential for the development of new drugs, at present it is increasingly under threat from commercial influences resulting in an urgent need for rules and guidelines to safeguard the reliability of trials. Bias in publishing positive results and under reporting of negative results was also highlighted.

**Ethical medical writers must always acknowledge the original source of the idea, text or illustration - Prof.Paolo Pozzilli**

**Mr. Shaukat Ali Jawaid** in his presentation on Scientometrics talked about Impact Factor, h-Index and other indexes. It was Eugene Garfield who first identified the importance of citations and then crated the Science Citation Index in 1950s. He then demonstrated how Impact Factor is calculated. He also talked about drawbacks of Impact Factor, the other alternatives like h Index, how it is calculated, advantages of h Index and its drawbacks. He also talked about m-Index, g-Index and e-Index and pointed out that each one of them can be manipulated and has its advantages and disadvantages.

**Mr. Tom Lang** from USA discussed what one should know how to get published. He said that one should ask oneself a question as to how will medicine be different if he or she answers research question? If you do not know the answer, it won’t be published. Ask yourself what problems needs to be solved or how something can be done faster, better, cheaper, safer and easier. What is known about the question? Has it already been answered? How have others tried to answer it? What variables were involved and how they were measured? What problems were found? Make the question specific i.e. How effective are medicated bandages? He further stated that science cannot exist without writing. Only writing allows science to be recorded, evaluated, reproduced, systematic, cumulative and public. Publication Tom Lang opined is the final stage of research and evidence based medicine is literature based medicine. The quality of medical care is affected by the quality of published articles. Follow the instructions for authors of the journal you are writing for was extremely important and will determine whether it is accepted for publication or not.

During the discussion it was stated that one should do something which need to be studied than what can be studied. One does not have to cure cancer but just find something that makes medicine better. If the topic selected is of local interest, such manuscripts cannot be published in an international journal. Editors want new research which is important and is clearly reported. One should ask timely and important questions. If the selection of the journal is wrong, topic selection is wrong, something has already been published on that topic and the journal has high Impact Factor, your manuscript will not be published.

**Dr. Bashair Musa** from Research Center for Diabetes and Research Ajman highlighted the factors affecting academic writing skills and mentioned time, motivation, language, texting culture, knowledge, training i.e. Underestimating the importance of good writing and disregard the principles of good writing. One should convey the thoughts clearly and professionally. Good communication skills, career progress, Research and Publishing are important. Always use full words, provide accurate detailed information, provide facts, numbers, never use abbreviation in title and first time write full with abbreviation in bracket and then one can use these abbreviations. Writing of course has to be learnt, he added.

**Dr. Amena Sadiya** from RCDR Ajman described UAE experience regarding conducting randomized controlled trials in clinical practice. Speaking about the planning phase in clinical trial she mentioned the hypothesis i.e. plans of the study, team formation for the study, study protocol and case report Form, budget and the ethics committee approval. In phase two the clinical trial should be registered in public database, prepare the essential documents, arrange for study medications, train the study team and then start subject’s recruitment. It is just like directing a Play. ICMJE in 2005 decided that no trial will be published unless it is registered in a public database. As regards informed consent of the patients, study subjects it is essential that they are explained everything in detail otherwise there will be drop out. Patients can be in control or in placebo group. The trial procedure should be strictly followed. Explain the possible benefits and adverse effects if any. The whole record should be kept confidential. The duration of the trial should be explained. The research subjects should have the liberty to withdraw at any time if they wish to and it must not affect the medical treatment. She further stated that the quality of data determines the quality of study. She also referred to missing data or feeding of wrong data which are some of the problems which are encountered sometime.

**Research subjects should have the liberty to withdraw at any time if they wish to and it must not affect the medical treatment-Dr. Amena Sadiya.**

Speaking about the reasons for non-compliance she mentioned the study subjects forget, development of side effects hence they do not want to continue. Some might change their mind to participate in the study. Short duration of study, simplicity of intervention like single dose drug, careful selection of study subjects who are likely to follow study protocol, explaining in detail about the study and what is expected from them and staying in contact with the participants will minimize the chances of non-compliance. She then talked about the termination procedure, breaking the code and monitoring possible toxicity. The evaluation phase consists of data management, statistical evaluation, finalization and archiving. It is extremely important the study should be reported and published even if the results are negative.

## How to Write Medico legal Reports

**Mr. Shaukat Ali Jawaid** from Pakistan made his presentation on How to write Medico Legal report and said that it comes under the domain of Forensic Medicine which is both an Art and a Science. Forensic Medicine is a branch of medicine which deals with application of principles of medical knowledge for the purpose of law and for furthering Justice.

Writing medico legal reports is a highly specialized job which should not be undertaken by some one who has no experience or training in Forensic Medicine. In case of difficulty or inability to frame opinion, it is advisable to refer the matter to senior colleagues who are well trained for advice. Final opinion in the medico legal report, he said, should contain comments about nature of causative agent, estimation of time lapsed, gravity of damage inclusive of incapacity produced by trauma or intoxication. The possibility of injury being self inflicted, homicidal or accidental should also be recorded. Working in the medico legal section of a healthcare facility is a very dangerous job and one must be careful of all these consequences which at times could be too harsh, he remarked.

The practice of forensic medicine Mr. Shaukat Ali Jawaid stated extends beyond the premises of hospitals into the courts of law in continuation of work which has already been done in the hospital to help further justice. A Medical Jurist has to present, authenticate, interpret and justify his work done in the hospital as factual and true. All this requires efficiency, precision, neatness and full concentration. Forensic Medicine work must reflect respect for human being victim or accused.

It is extremely difficult while dealing with dead bodies under putrefaction. Besides legal, moral consideration, the job may become hazardous endangering safety of workers while they are medically examining the injured or dead. As such it requires recognition of risk and identification of hazards beforehand. Medico legal certification is highly technical hence it should not be attempted half heartedly. Only trained, qualified forensic medicine examiners should perform and make their opinion reliable and creditable.

**Writing medico legal reports is a highly specialized job which should not be undertaken by some one who has no experience or trainingin Forensic Medicine - Shaukat Ali Jawaid**

Despite the fact that examination of living and dead are similar but it differs in technical details and status of the examinee. Autopsy is more specific with additional objectives i.e. determining cause and manner of death, estimation of fatal and postmortem periods. Medico legal examination requires purpose built centers supported with proper and specific implements in medico legal clinics for living and autopsy suits are required for the Dead.

Workload of forensic medicine includes physical and sexual assaults, intoxication resultant from self or criminal poisoning with drugs, accidental trauma on the road, rail, air and industry. Medical certificates may also be required for life insurance, police recruits, and drivers, age certification for schooling and fitness for job, marriage, election as well as capital punishment.

There are certain pre-exam formalities. In sexual assault, he said, one has to determine whether the injury has produced any damage to private parts of the victim, injury resulted in damage or defect in private parts. Information about weapons used, number of persons involved, circumstances i.e. place of occurrence is also extremely important in case of physical and sexual assault cases. In case of road traffic accidents, industrial accidents it is important to listen to the victim carefully and record history of occurrence. Of course professional ethics demands that one has to take consent of the victim before starting the examination.

Physical examination of clothes Mr. Shaukat Ali Jawaid remarked has a special place in forensic medicine. They are preserved, searched to locate any foreign material present on them i.e. Stain, hair, fiber, stains of blood, semen, vomitus, excreta, oil, its position, colour and distribution should all be recorded in the medico legal certificate. Details about smell, feel, shape and texture must also be recorded. One should use naked eye as well as magnifying lens. Any damage, cut in clothes is an important finding in forensic medicine. One should note its position and relationship to the injury. Examination should include physical exam, mental status examination, clinical systematic examination, local examination of the affected part. Local examination means exam of body parts or any portion with pathology, wound caused during assault or accident. Examination also includes exam of body opening violated during sexual assault. Wound margins, walls bed whether smooth, cleanly cut or lacerated needs to be carefully noted.

Body opening of vagina and anus if violated sexually must be examined. These openings are best examined in lithotomy and knee-elbow position respectively. Adequate illumination is essential for good results. In Forensic Medicine, solely relying on victim’s statement could be hazardous. As regards documentation and certification, written record, drawn sketches, photographs must be prepared and preserved. Close up photographs of injuries, damage, staining of clothes, full view of injured or intoxicated are all very essential. Fractures should be recorded with X-rays. The certificate should include result of investigations, reports on collected material and all other relevant documents such as receipts and dispatches. In case the injured is admitted to the hospital, treatment notes, duration of stay, date of discharge should be obtained and incorporated in certification to help verify full facts of the case. It is worthwhile to note that medical opinion or conclusions should only be based on observed facts. Opinions should not go beyond knowledge of the medical examiner. Sentiments, sympathy, personal theory must not influence the formulation of opinion. It is also essential to record whether the findings are consistent with sexual intercourse or otherwise. He also acknowledged the help and guidance of Prof. Naseeb R. Awan an eminent Forensic Medicine specialist from Pakistan for preparing this presentation.

**Dr. Nader Lessan** from UAE discussed writing the manuscript. One of the topics suggested by the participant for the study was why AMI is seen in young people in UAE. In this, one has to find out the reasons and also define what you mean by the young age. While writing on this topic, in the introduction one should give the global figures for AMI, its relevance, life style changes, risk factors in UAE, differences in prevalence in UAE and other countries and then justify why you wish to do this study. In the methods section, one can mention that it is a retrospective study for the last five years and the data in a particular hospital was looked at from Dubai Health Authority. The electronic data is used and the risk factors are reviewed. While writing the results, provide patients characteristics in the form of a table, it may also include figures and the overall number. Then you discuss your results with findings from other similar studies from UAE, from the region and globally in the discussion section and find out how and why they are different if at all.

On third day of the conference **Mr.Habibur Ibrahim** talked about pharmacy related open access journals from India. Open access encourages more citations. He also referred to the DOAJ which has two hundred journals from USA. During the Year 2011, 169 journals were included in DOAJ. He was of the view that one must check the validity of journals while looking at the open access journals.

Next presentation was on outsourcing medical writing services. It was pointed out that in this skilled medical writers are hired and they are cost effective and it also meets the guidelines. These professional writers work with mutual respect and trust. The sponsors of the study are always the owners of the data. In the beginning they sign agreement regarding confidentiality of study data. During the discussion it was pointed out that one should use editing services before making final submission. Universities and medical institutions use the services of professional medical writers because it is most cost effective.

**Markus Heinemann** from Germany gave highlights of cardiac surgery reflected in one journal over a period of thirty four years. This was based on MD thesis. The journal named Thoracic and Cardiovascular surgery was founded in 1953. It became a university journal in 1972 and in 1974 it was converted into English and included in ISI. In 1953 its all issues were put online and they publish eight issues every year. So far the journal has published 3350 articles and now 78% of its manuscripts are related to cardiac surgery. Almost 15% of the papers were on congenital heart disease. Case reports are very popular and they are covered in the last in the journal. During 208-2011 there has been an increase in publication of case reports. Experimental research work accounted for 15%. It was further stated that case reports are extremely important in surgery; they are widely read but hardly cited. Dr. Markus further stated that now they have started a case reports journal, almost 60% of the submitted articles are rejected and there is 75% rejection in the main journal. He concluded his presentation by quoting Ibne Sina who had remarked that we should try to know the cause of the disease.

**Sam Mathews** from India presented highlights of a decision support tool they have developed to determine the authorship in clinical publications which looked quite useful. During the discussion issues like listing of authors and credit criteria all figured prominently.

## Panel Discussion

The conference concluded with a panel discussion on Online Health Information. The panelists included **Tom Lang** from USA, **Julia Donnelly** from UK, **Shaukat Ali Jawaid** from Pakistan, **Farhad Handjani** from Iran and **Gamela Nasr** from Egypt. It was pointed out that each one of us should try to be a vehicle of change. All the information available on the net is not credible. Equator website has guidelines for reporting research findings. Then there is CONSORT statement for clinical trials. It was reiterated that one should carefully look at the credibility of the websites. Ethics Mr. Shaukat Ali Jawaid remarked has a lot to do with morality. Mr. Tom Lang said that at time manuscripts are rejected because of poor English and Grammar. Some of the journals are very large and some have part time staff that cannot help the authors. The easiest way is to reject such manuscripts because these journals do not have the resources to rewrite these manuscripts. Hence some commercial companies have come up who provide these services of improving English language. Some of these companies are very professional and do a quality job. It was also suggested that we should try to find an indigenous solution to indigenous problems which alone will be cost effective. Mr. Tom Lang said that he needs about seven to eight hours to correct a manuscript and it takes two three days. However some companies have acquired the services of professional writers who are asked to correct and edit fifty articles in a week hence how you can expect a quality job from them, he asked.

